# Post-surgical Outcomes of Different Surgical Techniques in Hirschsprung’s Disease: A Literature Review

**DOI:** 10.7759/cureus.47012

**Published:** 2023-10-14

**Authors:** Pragathi Munnangi, Anam Sayed Mushir Ali, Sheryl Deva, Varsha Kushwaha, Shivangi Srivastava, Aishwarya Boini, Ritu S Agarwal, Prateek Kumar Dinkar, Esha Chaudhary

**Affiliations:** 1 Medicine, NRI Medical College, Guntur, IND; 2 Medicine, Indian Institute of Medical Science and Research, Aurangabad, IND; 3 Medicine, Kamineni Academy of Medical Sciences and Research Centre, Hyderabad, IND; 4 Medicine, Grant Government Medical College and Sir JJ Group of Hospitals, Mumbai, IND; 5 Medicine, TS Mishra Medical College and Hospital, Lucknow, IND; 6 Internal Medicine, Davao Medical School Foundation, Davao, PHL; 7 Internal Medicine, DY Patil University School of Medicine, Navi Mumbai, IND; 8 Emergency Medicine, King George's Medical University, Lucknow, IND; 9 Internal Medicine, Government Medical College, Kannauj, IND

**Keywords:** laparoscopic-assisted tept, transanal endorectal pull-through (tept), pediatric gastroenterology, pediatric surgery, laparoscopy, postoperative outcomes, hirschsprung disease (hd)

## Abstract

Hirschsprung's disease (HD) is a rare condition that affects newborns and is characterized by the lack of ganglion cells in the colon. Typical symptoms include difficulty passing stool, vomiting, and trouble feeding. Various surgical methods are available to manage the condition. The aim of the study is to investigate and compare the post-surgical outcomes of different surgical techniques used in the treatment of HD. A thorough literature search was conducted using various electronic databases to identify relevant studies to be referred to. Double-blinded screening of the identified articles led to the final selection of 40 out of 440 HD, including transanal endorectal pull-through (TERPT), laparoscopic approaches, and modified techniques. Several studies have investigated surgical procedures for HD, including TERPT, laparoscopic methods, and modified techniques. These have shown positive outcomes, with fewer complications, improved bowel function, and favorable cosmetic results. Individual patient characteristics and surgeon expertise should guide procedure selection. Surgery for HD aims to restore normal bowel function, but post-surgical outcomes can include constipation or fecal incontinence. Complications like enterocolitis, anastomotic stricture, and sphincter damage may occur. Laparoscopic approaches have shorter hospital stays. However long-term follow-up is essential to assess quality of life, psychological well-being, and potential side effects.

## Introduction and background

Hirschsprung’s disease (HD) also referred to as aganglionosis, is distinguished by the lack of ganglion cells within the myenteric and submucosal plexuses found in the colon. The absence of ganglion cells in this particular segment leads to a condition where the intestinal contents cannot move effectively, causing stagnation or lack of contraction in that segment [1,2]. HD is found in approximately one out of every 5,000 live births and is commonly diagnosed during the neonatal period. During this time, affected individuals exhibit symptoms such as a swollen abdomen, inability to pass meconium, expulsion of bile through regurgitation, vomiting, and difficulty with feeding. The most effective and used method for diagnosing HD is rectal biopsy, which can be performed by obtaining a full-thickness specimen or by suction technique obtaining a full-thickness specimen [3]. This diagnostic approach is the gold standard as it is confirmed by demonstrating the absence of ganglion cells [4]. 

The surgical management of HD involves employing various techniques. These techniques are widely practiced worldwide and commonly include procedures such as Swenson, Duhamel, and Yancey-Soave. Currently, the two most frequently performed surgical procedures to treat rectosigmoid HD are endorectal pull-through (ERPT), which includes both total transanal (TERPT) and laparoscopic endorectal pull-through (LERPT) approaches. In this technique, our objective is to surgically remove the affected segment and bring down the normally innervated bowel [5]. Pediatric surgeons follow children with HD from diagnosis to corrective surgery in infancy, to check for and manage short-term and long-term complications. Typically, HD primarily impacts a limited section of the large intestine or colon. The rectum is consistently implicated, with 80% of instances confined to the rectosigmoid colon. However, in rare cases, certain individuals may experience a complete absence of ganglion cells throughout the entire colon [6].

It has been reported that up to 60% of children have complications after corrective surgery [7]. In the immediate postoperative period, patients report fecal soiling and diarrhea unrelated to obstruction, which typically normalizes over several months [8]. During this transition, young children may also experience severe perianal excoriation. Long-term obstructive complications include anastomotic stricture, achalasia of the anal sphincter, residual aganglionosis, fecal incontinence of varying degrees, or recurrent enterocolitis. Several meta-analyses have previously examined the immediate and intermediate postoperative results in individuals with HD who received various surgical methods. However, the incidence of postoperative complications remains inconsistent [9-13]. 

Quite a few studies have been published, which include studies to evaluate outcomes of surgery for HD in the pediatric population. Our aim is to conduct a systematic review of existing literature to summarize the range of immediate and long-term complications and outcomes in various surgical procedures for HD to ease evidence-based decision-making for choosing a surgical option for the treatment of HD.

## Review

Methodology

**Figure 1:**
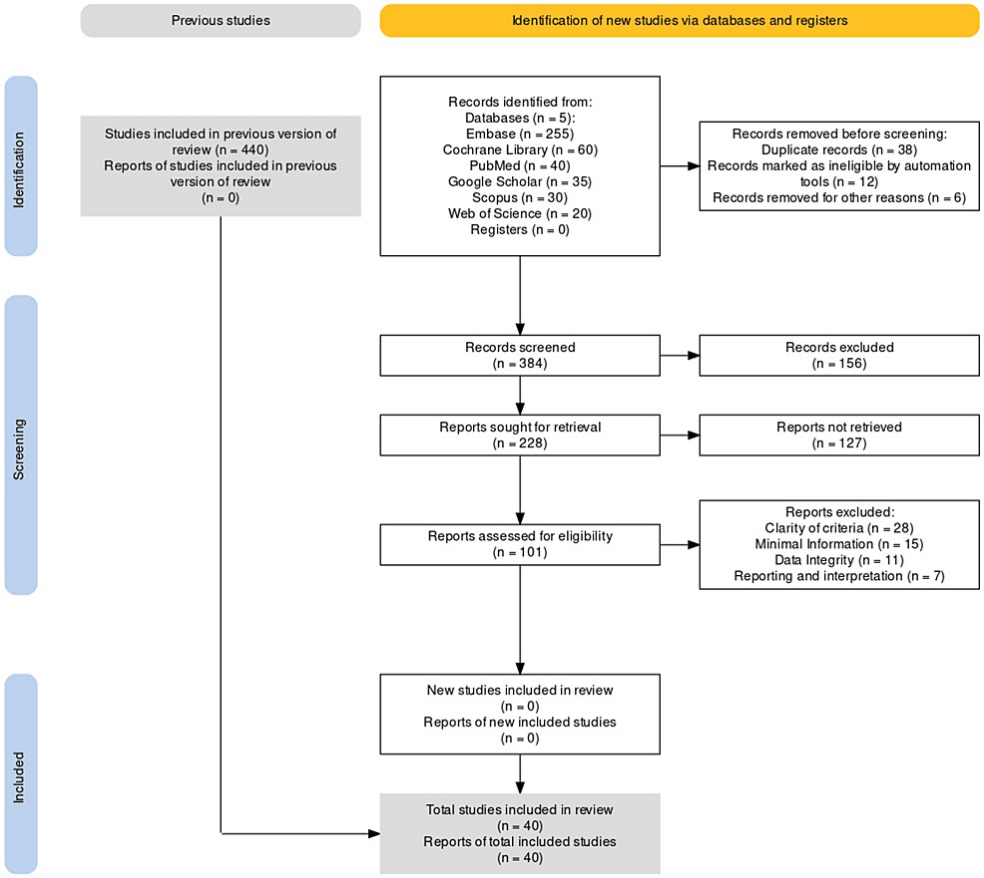
PRISMA flow chart with the description of studies inclusion PRISMA: Preferred Reporting Items for Systematic Reviews and Meta-Analyses

The literature search was conducted using a systematic approach to identify relevant studies for inclusion in this review. The following electronic databases were searched: Embase, Cochrane Library, Google Scholar, PubMed, Scopus, and Web of Science. The search strategy combined relevant keywords and Boolean operators to ensure comprehensive coverage of the literature. The search terms used in the strategy included “HD,” “postoperative outcomes,” “laparoscopy,” “pediatric surgery,” “pediatric gastroenterology,” “transanal endorectal pull-through (TERPT),” and “laparoscopic-assisted transanal endorectal pull-through (LTEPT).” The studies involving sigmoid volvulus were included in the literature search, and other volvulus cases (cecal, transverse, or splenic) were excluded. These references were meticulously reviewed, and key findings were extracted to create a comprehensive literature review. The search was limited to articles published in English between 2023 and 2010. No other restrictions, such as publication status or study design, were applied. The initial search yielded a total of 440 articles. A double-blinded screening was applied and a total of 40 articles were included in this literature review (Figure 1). Additionally, reference lists of identified articles and relevant reviews were manually searched to identify any additional studies that might have been missed in the electronic search.

HD is a congenital condition of the gastrointestinal tract characterized by the absence of ganglion cells in the distal bowel and has been referred to as a congenital aganglionic megacolon.

Surgical techniques

Several surgical techniques have been developed in recent years. Removing the aganglionic portion of the gastrointestinal tract and restoring normal bowel continuity is an essential objective of surgery.

Factors such as the length of the affected bowel section, associated abnormalities, and surgeon experience shall be taken into account when choosing an approach. In recent years, minimally invasive techniques, such as laparoscopic and robotic-assisted approaches, have gained popularity due to their potential advantages, including reduced postoperative pain, shorter hospital stays, and improved cosmetic outcomes [2,14-19]. The TERPT and LERPT are the predominant surgical techniques utilized for the treatment of rectosigmoid HD [5]. Accurate preoperative bowel preparation, correct trocar placement, and patient positioning proved to be crucial aspects of treatment [20]. Surgical intervention to remove the affected portion of the stomach is an important aspect of the final treatment for HD to restore normal bowel function. The objective of this discussion is to present a comprehensive review of surgical outcomes in pediatric patients with HD.

Preoperative evaluation

A thorough preoperative evaluation, including a detailed medical history, physical examination, and diagnostic tests, is the first step in the management of HD. The evaluation of clinical characteristics, age at diagnosis, associated comorbidities, and nutrition status will be a major part of the assessment. There were no significant differences in the diagnosis or occurrence of enterocolitis, and the proportion of patients requiring bowel management for fecal incontinence was also similar [21,22]. In a study by Lin et al., it was found that preoperative anal dilatation did not show any significant difference in outcome when compared to patients with no preoperative anal dilatation; hence, improving preoperative maneuvers might not help improve postsurgical outcomes for HD [23]. A conclusion was drawn that there is no significant difference in effect on growth outcomes among different procedures [24]. The study by Onishi et al. stressed the positive effects on children's quality of life by resolving post-operative issues through medical treatments or redo procedures [25]. Moreover, to confirm the diagnosis, assess the size of the aganglionosis, and decide how to proceed with surgery, it is also necessary to use different diagnostic methods like contrast enemas, rectal suction biopsies, or anorectal manometry [26]. In a study by Ralls et al., redo pull-through patients of HD have worse stooling results than initial operations [27]. A study analyzed the growth outcomes of patients who underwent different pull-through methods; the Duhamel procedure (DP), Swenson procedure (SP), and TERPT showed the improvement of nutritional status was achieved in 21.2% of HD patients after TERPT, 14.3% post-Duhamel, and 5.9% following the Soave procedure [28]. In a study by Urushiara et al., it was shown that laparoscopic Z-shaped colorectal anastomosis for HD appears feasible and safe to perform with good results [29].

Short-term outcomes

Short-term outcomes following HD surgery are crucial for immediate recovery. Key measures include surgical complications, pain, oral intake time, hospital stay, and bowel function. A study by Xu et al. comparing transumbilical enterotomy and conventional abdominal enterostomy found no significant difference in soiling and constipation rates. However, the transumbilical approach showed better cosmetic results [1].

Surgical complications, although rare, can occur and range from wound infections and anastomotic leaks to enterocolitis and obstructive symptoms. In a study by Askarpour et al., children who underwent Saove's pull-through procedure with oblique and circular anastomosis were followed up for soiling, and postoperative complications, such as wound infection, wound dehiscence, peritonitis, fecal soiling, and perianal excoriation, were recorded for each patient [17]. The study found that perianal excoriation was the most common complication among patients in both groups. Oblique anastomosis yielded fewer complications than circular anastomosis, making it a viable choice for patients who underwent Soave's procedure. Also, enterocolitis was more frequent in the circular group than in the oblique group. The anastomotic stricture was also more frequent in the circular group [17]. Anorectal function generally improves with age. In contrast, early postoperative complications may involve perianal excoriation, enterocolitis, and an anastomotic leak, while late postoperative complications can include issues such as anal stricture, constipation, and soiling problems [30-32]. Using a transcolostomy, a single-incision laparoscopic approach provides a cosmetic benefit by reducing scarring. In addition, it provides surgical accuracy, reducing rates of intestinal perforation and, thus, reducing the incidence of postoperative intestinal obstruction caused by adhesions. Preventing tension between the anal stoma and colon is crucial to reduce complications like anal stenosis and enterocolitis, while a comparison between the SP and DP reveals that the SP has a higher frequency of bowel movements and more severe soiling but lower constipation severity [33-35]. In a study by Kastenberg et al., patients who underwent primary endorectal pull-through for HD were analyzed, and the outcomes of neonatal and delayed pull-through cohorts were compared and found that delayed pull-through was a safe alternative to neonatal surgery with similar functional outcomes [35].

A study by Dingemans et al. indicated that a primary laparoscopic endorectal pull-through procedure with a postoperative rectal tube reduces early-stage abdominal distension and Hirschsprung-associated enterocolitis (HAEC), offering beneficial postoperative management [36].

Postoperative complications in the surgical management of HD, such as HAEC, fecal incontinence, perianal excoriation, anastomosis stricture and leakage, anastomosis volvulus, and anovaginal fistula, vary in frequency based on the specific surgical procedure [21, 37]. TERPT has positive outcomes, with shorter hospital stays and fewer postoperative complications.

However, long-term complications such as constipation and soiling occur more frequently due to rectum mobilization and anal sphincter stretching. Certain measures such as leaving the rectal cuff short after TERPT and leaving the native rectum short after Duhamel pull-through do help to reduce the incidence of constipation postoperatively. Rectal stenosis, which occurs due to anastomotic ischemia, and anastomotic leakage were lower following the Duhamel pull-through [38]. The significant factor for long-term complications was whether HD was associated with a syndrome or not. Between the two, the transanal pull-through procedure is preferred over the Duhamel procedure.

Long-term outcomes

Long-term surgical outcomes in HD focus on restoring normal bowel function. Most patients achieve satisfactory results, improving their quality of life. However, some may experience issues like fecal incontinence and constipation. Factors such as segment length, genetic syndromes, comorbidities, and surgical technique can influence outcomes. Fecal soiling is a common concern during long-term follow-up, which can be minimized by proper positioning of the mucosectomy incision on the anal canal [24].

Modified laparoscopic Swenson (MLSw) and laparoscopic Soave (LS) procedures when compared showed that early postoperative outcomes like soiling and constipation were much better in the MLSw group, but long-term outcomes were similar in both procedures [18,26]. Reoperations to treat complications showed symptomatic improvement.

Quality of life

Constipation was stated as the predominant concern by almost 15% of patients having TERPT [15]. The transanal approach was praised for being less intrusive and having fewer problems than the Swenson-Denda (SD) treatment [16]. The study by Oh et al. investigated stool frequency and soiling in individuals with HD [34]. The findings showed that shorter aganglionic segments were correlated with reduced bowel movements and milder soiling. However, as patients aged, these distinctions became less prominent [34]. 

Surgical intervention remains crucial in treating HD, providing relief from symptoms, promoting growth, and improving function. However, long-term follow-up is essential to assess the quality of life, psychological well-being, and potential side effects. It is important to investigate factors influencing outcomes and establish standardized follow-up protocols for patients with HD and other conditions.

Significant findings from specific studies

Pini Prato et al. substantiated the minimally invasive redo pull-through technique's appropriateness for treating postoperative obstructive problems [20] (Table 1). Oblique anastomosis in the Soave pull-through method has the potential to reduce postoperative problems as compared to circular anastomosis. When compared to the Duhamel procedure, the Swenson procedure had a higher frequency of bowel movements and a higher severity of soiling [22]. In a two-stage technique, Xu et al. indicated good outcomes and enhanced cosmetic effects of transumbilical enterostomy (TUE) compared to conventional abdominal enterostomy (CAE) [1].

**Table 1:** Table showing the postoperative outcome in HD in different studies HD: Hirschsprung's disease LoHs: Length of hospital stays TUE: Transumbilical enterostomy LERPT: Laparoscopic endorectal pull-through TERPT: Transanal endorectal pull-through CS: Corrective surgery DD: Defecation disorder TA: Transanal LAPT: Laparoscopic-assisted pull-through LATP: Laparoscopic-assisted transanal pull-through CTP: Complete transanal pull-through RSPT: Robotic Soave pull-through MIRPT: Minimally invasive redo pull-through TRSPT: Totally robotic soave pull-through HAEC: Hirschsprung-associated enterocolitis SD: Soave-Denda PT: Pull-through TAB: Transabdominal approach LTD: Laparotomic Duhamel LSD: Laparoscopic Duhamel LTEPT: Laparoscopic-assisted transanal endorectal pull-through NA: Not available

Author, year	Study design	Sample size	Surgical technique	Postoperative complications	Hospital stay LoHs	Outcomes	Follow-up	Complications after follow-up	Other noteworthy findings
Xu et al., 2019 [1]	Retrospective observational	53	Transumbilical enterostomy in two-stage laparoscopy-assisted pull-through compared with conventional abdominal enterostomy	Stomal mucosal prolapse, wound infection, obstruction, enterocolitis	10 days	TUE approach demonstrated favorable results, and the cosmetic outcome	Not specified	Not specified	TUE achieved a similar cosmetic effect to one-stage laparoscopy on the abdominal wall
Menon et al., 2022 [2]	Retrospective analysis	28	Laparoscopy-assisted pull-through. 26 patients underwent Swenson, 1 underwent Soave, and 1 underwent Duhamel procedures	NA	6 days	Laparoscopic-assisted pull-through is a safe and feasible option in pediatric patients with a considerably low risk of complications	3 weeks	Enterocolitis, night-time soiling, constipation	Laparoscopic-assisted pull-through is a safe and feasible option in pediatric patients with a considerably low risk of complications
Ahmad et al., 2022 [3]	Review study	NA	Evaluation and treatment of the post-HD pull-through patient who is not doing well	1) Fecal incontinence, (2) obstructive symptoms, and (3) recurrent episodes of enterocolitis	NA	A thoughtful, systematic approach will help the clinician to determine the cause of the problem and adopt a successful therapeutic plan to manage postoperative complications of HD	NA	NA	Patients may need medical management (behavioral interventions, dietary changes, laxatives, or mechanical emptying of the colon), a reoperation when a specific anatomic or pathologic cause is identified, or botulinum toxin when non-relaxing sphincters are the cause of the obstructive symptoms or recurrent enterocolitis
Teitelbaum & Coran, 2003 [4]	Review study	NA	NA	NA	NA	Primary pull-through in the neonatal period is an acceptable procedure for HD	NA	NA	NA
Karlsen et al., 2022 [5]	Cross-sectional study	91 (46 TERPT and 45 LERPT)	Comparison of clinical outcomes after TERPT and LERPT	Perineal excoriation, gastroenteritis, wound infections, obstructive symptoms, enterocolitis, anastomotic structure, and leakage	6 days	No difference in long-term bowel function in patients operated with TERPT and LERPT	30 days	26% of TERPT and 32% of LERPT reported fecal soiling; constipation	Patients operated on in the neonatal period had poorer outcomes (78%) than those operated on later (24%) through TERPT
Green et al., 2016 [6]	Review article	NA	Swenson, Duhamel, and Soave pull-through procedures	Fecal soiling, diarrhea	NA	Management of postoperative complications is required	NA	Obstruction	Preoperative daily rectal irrigation or placement of ostomy helps to prevent enterocolitis
Chumpitazi & Nurko, 2011 [7]	Retrospective review	57	Addressed protracted defecation disorders in children with HD after corrective surgery (CS)	Enterocolitis, obstructive symptoms, fecal incontinence, constipation	NA	The majority of children with HD and DD after CS have favorable long-term clinical outcomes	41.4+-4.5 months	Fecal incontinence	Children with enterocolitis were more likely to have excellent or good clinical outcomes after the second surgical intervention
Langer et al., 2013 [8]	Review article	NA	Outcomes of various pull-through procedures	Frequent stools, perianal skin breakdown, enterocolitis	24-48 hrs	Enterocolitis and obstructive or soiling resolves in most of the cases with appropriate diagnosis and treatment over time	NA	Obstruction and soiling	None
Zimmer et al., 2016 [9]	Meta-analysis	316	Transanal pull-through	Constipation (53.3%), incontinence/soiling (17.8%), enterocolitis (28.9%)	NA	Nearly 15% of all patients operated with TERPT experience constipation as the main problem	3 years	Constipation, soiling	NA
Zhang et al., 2015 [10]	Meta-analysis	774	Laparoscopic-assisted operations and laparotomy for HD	NA	2.7 days	Compared with laparotomy operations, laparoscopic-assisted operations are generally safer and more reliable for patients with HD.	NA	NA	NA
Thomson et al., 2015 [11]	Systemic review and meta-analysis	405	Comparison of outcomes of total TERPT vs LAPT	Enterocolitis, fecal incontinence constipation unplanned laparotomy, or stoma formation injury to abdominal viscera	NA	No evidence of a higher rate of enterocolitis incontinence or constipation following total TERPT compared to LAPT	NA	Intestinal obstruction intestinal ischemia enteric fistula formation urinary incontinence or retention	NA
Guerra et al., 2016 [12]	Retrospective review	24	Laparoscopic-assisted transanal pull-through (LATP) vs complete transanal pull-through (CTP)	NA	NA	Operative time was significantly longer for the LATP group (OR 1.59, 95% CI 1.21-1.96, p<0.001)	NA	NA	No significant difference in major complications (OR 1.75, 95% CI 0.76-4.04, p=0.19) or length of stay (OR 0.33, 95% CI -0.41 to 1.08, p=0.38).
Dai et al., 2020 [13]	Systematic review and meta-analysis	625	Soave, Duhamel, Sphincteromyectomy, Ileostomy, Sigmoid colostomy, transanal endorectal pull-through (TERPT), TERPT with laparotomy/laparoscopy, Ileoanal pull-through; definitive endostomy. Duhamel-Martin modification, Ileorectal anastomosis	NA	NA	For patients older than ten years with an HD surgical history, the prevalences of fecal incontinence, constipation, and bladder dysfunction symptoms are 20, 14, and 7%, respectively; and these patients generally have lower gastrointestinal quality of life index compared to healthy population	NA	Fecal incontinence, constipation, urinary system dysfunction	NA
Delgado-Miguel & Camps, 2022 [14]	Prospective study	15	Robotic Soave pull-through (RSPT) procedure	Partial anastomosis dehiscence (n=1)	Median hospital stay- 3 days (interquartile range 3-4)	RSPT procedure for HD in children younger than 12 months is a safe and effective procedure	79 months (interquartile range 45-115)	Constipation (n=2) mild enterocolitis (n=1)	NA
Gandhi et al., 2022 [15]	Retrospective review	12	TERPT	Perianal excoriation (n = 2) and enterocolitis (n = 1)	Mean - 8 days	Effective technique in the management of HD with minimal complications	2 years	Increased stool frequency	NA
Askarpour et al., 2021 [17]	Retrospective analysis	70	Saove's pull-through procedure with oblique and circular anastomoses	Early complications of Soave’s procedure include anastomotic leak, peritonitis, pelvic infection, septicemia	7± 2.1 (oblique type), 8 ±2.7 (circular type)	Oblique anastomosis can reduce postoperative complications in contrast to circular anastomosis	Duration - 2 yrs after surgery	Strictures, enterocolitis, mucosal prolapse, incontinence, and perianal excoriation	No significant difference between the two groups in terms of wound infection, length of hospital admission, bleeding during operation, and length of surgery
Pini Prato et al., 2020 [19]	Prospective study	16	Minimally invasive redo pull-throughs in HD	NA	49 months	MIRPT proved to be effective and safe in HD patients complaining of postoperative obstructive symptoms.	NA	Cuff stricture, enterocolitis	NA
Pini Prato, et al., 2020 [20]	Prospective study	11	Totally robotic soave pull-through (TRSPT)	mild postoperative enterocolitis	NA	TRSPT is particularly suitable for older HD patients, even those requiring a redo	12 months (median)	NA	NA
Imvised et al., 2016 [21]	Literature search	76	TERPT	HAEC, intestinal perforation, incontinence, perianal excoriation, perianal stricture, anastomotic leakage, retained aganglionic segment, anovaginal fistula	NA	TERPT is safe and feasible in all pediatric age group	NA	NA	NA
Hashim et al., 2018 [22]	Descriptive case series	75	Modified Duhamel’s operation	Enterocolitis in 50% of patients. 8 children had surgical site infection. Anatomic stricture occurred in 14.7 %. Intestinal obstruction -10.3% Constipation -16%	Not specified	The modified Duhamel Procedure is quite safe. It has fewer postoperative complications	NA	NA	NA
Lin et al., [23], 2021	Prognosis study	95	Anal dilatation before surgery	NA	NA	Operative time reduced, risk of developing postoperatively HAEC reduced, postoperative obstructive symptoms reduced	NA	NA	NA
Kawaguchi et al., 2021 [24]	Systematic review	449	Duhamel, Martin, Soave/Swenson, right colon patch (Kimura) approaches	HAEC is common for the long segment and total colonic HD-31-80%. More soiling and incontinence in patients with long-segment HD	NA	Increased long-term gastrointestinal symptoms in long-segment HD	NA	N9A	NA
Onishi et al. [25], 2016	Retrospective review	110	Comparison of transanal endorectal pull-through (TERPT) and the Soave-Denda (SD) procedure.	In the SD group, anastomotic leakage (4.3%), cuff stenosis (14.5%) enterocolitis (17.4%) were observed. In the TERPT group, anastomotic stricture (0%), cuff stenosis (2.7%), and enterocolitis (10.8%) were noticed	In the SD group, hospital stay was 23.93±8.28 days compared to the TA group where it was 17.78±7.23 days	TERPT procedure is minimally invasive and has fewer complications when compared with SD	NA	The incontinence score at 3, 9, and 11 years of age was lower in the TA group than in the SD group	NA
Neuvonen et al., 2017 [26]	Cross-sectional study	123 patients and controls	Transanal endorectal pull-through (TERPT)	Holding back defecation, fecal soiling, and fecal accidents occurred in 9%, 25%, and 11% of patients	Not specified	Postoperative complications improve with age	Not specified	Abnormal frequency of stooling persisted in adulthood in 50%	NA
Ralls et al., 2014 [27]	Retrospective review	46	Compare Redo PT and primary pull-through	Primary pull-through: Early complications included anastomotic leak (19%), obstruction (9%), twisted PT (4%), and enterocolitis (6%). Late complications we're obstructive symptoms. In Redo pull-through, constipation was common	NA	Patients requiring redo PT have worse stooling outcomes compared to those after a primary PT	6.3– 491.6 months	NA	NA
Ksia et al. [28], 2013	Retrospective review	20	Soave one-stage endorectal pull-through procedure	Early complications included ileo-ileal intussusception and anastomotic leak. Late complications were Irritation of the perineum in 25%, anal stenosis in 20%, and Postoperative enterocolitis in 5%	3 to 10 days	In children above two years of age, Soave transanal one-stage endorectal pull-through was found to be safe and with low morbidity and mortality	NA	NA	NA
Urushihara et al., 2012 [29]	Review	26	Laparoscopic modified Duhamel procedure comprising Z-shaped anastomosis	NA	NA	This technique produced good results of normal defecation without fecal incontinence	50.4 months	84% showed episodes of constipation during the early follow-up period	NA
Zani et al., 2017 [30]	Retrospective review	294	Soave in 65% of patients, Swenson (19%), and Duhamel approach (16%)	NA	NA	The most common technique used for PT is the transanal Soave for patients with standard segment HD	NA	NA	NA
Yang et al. 2012 [31]	Retrospective review	137	Modified transanal procedures	Perianal excoriation (27.7%), enterocolitis, anastomotic leak	5–16 days	The defecation function was satisfactory in our long-term follow-up	6 months–9 years	94.8 % had excellent/good to fair fecal continence	NA
Deng et al., 2015 [32]	Systematic review	625	Duhamel and Soave methods, Transanal endorectal pull-through (TERPT), Swenson, State-Rehbein	Fecal incontinence, constipation, and bladder dysfunction symptoms are 20, 14, and 7%, respectively	NA	Most patient's gastrointestinal health-related quality of life is not as good as their healthy peers	10 year follow-up	NA	NA
Dutta HK, 2010 [33]	Retrospective review	45	Conventional TERPT procedure vs modified TERPT	Anastomotic stricture or stenosis, partial anastomotic dehiscence seen in conventional TERPT and not in modified TERPT	8 days	Modified TERPT procedure has fewer post-operative complications	36 months in TERPT group and 32 months in group modified TERPT	NA	NA
Oh et al., [34], 2020	Retrospective analysis	396	Pull-through (PT)	NA	NA	Compared to the DP, the SP was associated with an increased frequency of bowel movements and soiling severity; however, the constipation severity was lower	15 years of age	Fecal soiling, constipation	NA
Kastenbrerg et al., 2021 [35]	Retrospective study	82	Delayed primary endorectal pull-through	Fecal Incontinence, enterocolitis	NA	Delayed primary endorectal-pull-through is a safer alternative for neonates as it has fewer complications and equivalent functional outcomes	NA	NA	NA
Seo et al., 2018 [38]	Meta-analysis	430	Duhamel pull-through, TERPT	Constipation, incontinence, enterocolitis, anastomotic stricture, anastomotic leak	NA	NA	NA	NA	NA
Yan et al., 2019 [39]	Literature search	724	TERPT, transabdominal approach (TAB)	Incontinence, constipation, enterocolitis	Mean 6.74 days	TERPT is superior to TAB in terms of hospitalization time, postoperative incontinence, and constipation	NA	NA	NA
Heinrich et al. [40], 2011	Prospective study	8	All (re-operation for HD)	Stool incontinence, soiling	NA	Most complications leading to re-procedure of surgery are preventable	Mean 4.4 years	Soiling, stool incontinence	NA
Giuliani et al, 2011 [41]	Retrospective study	70	Duhamel, laparoscopic Duhamel, TERPT	Enterocolitis, constipation	Mean 4.4 days for TERPT, mean 6.8 days for LSD, mean 9.7 days for LTD	TERPT has better outcomes than Duhamel	NA	NA	Patients who underwent LTEPT had the earlier start of feeding and had a lesser hospital stay
Zhang et al., 2020 [42]	Retrospective et al	383	Primary laparoscopic endorectal pull-through procedure with or without rectal tube	Abdominal distention, HAEC	NA	Primary laparoscopic endorectal pull-through procedure with a rectal tube can reduce early-stage postoperative complications	Mean 2.0+0.53 days	NA	NA
Granstrom et al., 2013 [43]	Prospective study	27	Laparoscopic-assisted pull-through	Constipation, incontinence, loose stool soiling, soiling from solid stools	NA	Reduction of constipation	Between 2009-2012	Soiling, constipation, incontinence	High risk for incontinence, few signs of short-term improvement

The preoperative anal dilatation contributed to decreased operational time, postoperative HAEC, and obstructive symptoms. The majority of children spent between 5 and 8 days in the hospital. The most prevalent postoperative complications were enterocolitis, followed by fecal incontinence, constipation, and other difficulties such as anastomotic leak, perianal excoriations, blockage, and more.

Specific conclusions came from the remaining studies

There was no significant difference in long-term bowel function between TERPT and LERPT in research comparing the two procedures [5,10,11]. In terms of hospitalization length, postoperative incontinence, and constipation, TERPT surpassed transabdominal pull-through (TAPT). Overall, the quality of life of patients postoperatively depends on the level of self-esteem in children, which is influenced by the parental response to the child's illness [39]. Follow-up after redo surgery showed no complaints of constipation, stenosis, or intestinal obstruction, but soiling and fecal incontinence were reported at higher rates [29,40]. In a study by Giuliani et al., lighter impact of the surgical procedure on infants, a lower incidence of complications, and a better long-term outcome of the transanal pull-through compared to the Duhamel approach [41]. Zhang et al. showed that HAEC, which occurs due to intestinal obstruction and abnormal microbial flora, is another common complication that can be reduced by using a postoperative rectal tube after a primary laparoscopic endorectal pull-through procedure [42]. There is, however, a higher risk of incontinence after laparoscopy with fewer signs of short-term improvement [43]. The disorder predominantly impacts males with a ratio of about 4 males to 1 female being affected [44]. Congenital malformations contribute to around 7% of neonatal fatalities and result in a global burden of 25.3-38.8 million disability-adjusted life-years (DALYs) [45]. The relationship between HD and Down's syndrome is well-known, demonstrating an occurrence of 7.32% [46].

Importantly, even after a successful operation, individuals might have consequences such as enterocolitis, night-time soiling, constipation, and others. Impaired bowel control, infections, scar tissue development, and a weakened blood supply can all lead to these issues. Regular follow-up exams, imaging investigations, and coordination with specialists are required to address these difficulties.

Limitations and drawbacks

The possibility of publication bias exists in this literature review, wherein studies that demonstrate positive or significant outcomes are more likely to be published, while studies with negative or no significant findings might be underrepresented. This bias has the potential to influence the overall findings and could lead to an overestimation of the effectiveness of surgical outcomes. The included studies in the systematic review may have utilized different study designs, including retrospective and prospective designs, leading to heterogeneity in the data. Variability in patient populations, surgical techniques, follow-up durations, and outcome measures can affect the ability to draw definitive conclusions

The quality of the individual studies included in the review can vary. Some studies may have a higher risk of bias due to methodological flaws, inadequate sample sizes, or incomplete reporting of outcomes. The inclusion of lower-quality studies may introduce uncertainty or bias into the overall findings. Some studies may have incomplete or insufficient reporting of relevant data, such as complications, long-term outcomes, or patient characteristics. The lack of standardized reporting across studies can make it challenging to obtain a comprehensive overview of surgical outcomes.

The duration of follow-up in the included studies may vary, and there might be a lack of long-term follow-up data. This limitation hinders the assessment of late complications, functional outcomes, and the durability of surgical interventions. The review might be limited to studies published in English, which are available only in indexed journals. This restriction can introduce language and publication bias, potentially excluding relevant studies that were not accessible or included.

The review may not always account for confounding factors that could influence surgical outcomes, such as patient co-morbidities, surgeon experience, or the presence of additional congenital abnormalities. The absence of comprehensive data on these factors can limit the accuracy of the conclusions drawn. The review may be limited to studies published between 2023 and 2010. Older studies may not be included, potentially affecting the relevance of the findings.

## Conclusions

Surgery for HD aims to restore normal bowel function, and most patients achieve good or excellent bowel control post-surgery. TERPT is a preferred technique for HD, replacing staged procedures. TERPT and LERPT are commonly performed for rectosigmoid HD. Post-surgical outcomes include issues like constipation or fecal incontinence. Complications such as perianal excoriation, enterocolitis, anastomotic stricture, leakage, wound infection, and anal stenosis can occur but vary in frequency. Prompt recognition and treatment of enterocolitis are crucial. Achieving continence is important, but some patients may have persistent problems. The length of hospital stay varies depending on the procedure, with laparoscopic approaches generally associated with shorter stays. Enterocolitis is a common complication. Long-term sphincter damage may occur with TERPT. Single-incision laparoscopic surgery (SILS) with Soave TERPT offers cosmetic benefits and faster recovery. Post-surgical outcomes are influenced by various factors, and personalized information from healthcare professionals is essential.
